# Distress, anxiety, and depression in cancer patients undergoing chemotherapy

**DOI:** 10.1186/1477-7819-4-68

**Published:** 2006-09-26

**Authors:** Manoj Pandey, Gangadharan P Sarita, Nandkumar Devi, Bejoy C Thomas, Badridien M Hussain, Rita Krishnan

**Affiliations:** 1 Department of Surgical Oncology, Regional Cancer Centre, Trivandrum, India; 2 Department of Surgical Oncology, Institute of Medical Sciences, Banaras Hindu University, Varanasi, India; 3 Department of Psychology, HH Maharaja College for Women, Trivandrum, India; 4 Department of Psychosocial Oncology, Tom Baker Cancer Centre, Calgary, Alberta, Canada; 5 Department of Medical Oncology, Regional Cancer centre, Trivandrum, India

## Abstract

**Background:**

Chemotherapy for cancer is an intense and cyclic treatment associated with number of side-effects. The present study evaluated the effect of chemotherapy on distress, anxiety and depression.

**Patients and methods:**

A total of 117 patients were evaluated by using distress inventory for cancer (DIC2) and hospital anxiety and depression scale (HADS). Majority of the patients were taking chemotherapy for solid tumors (52; 44.4%).

**Results:**

The mean distress score was 24, 18 (15.38%) were found to have anxiety while 19 (16.23%) had depression. High social status was the only factor found to influence distress while female gender was the only factor found to influence depression in the present study.

**Conclusion:**

The study highlights high psychological morbidity of cancer patients and influence of gender on depression. Construct of distress as evaluated by DIC 2 may have a possible overlap with anxiety.

## Background

Treatment of cancer is by three main modalities namely surgery, radiotherapy and chemotherapy. The hematological malignancies and lympho-prolifaretive disorders are mainly managed by chemotherapy while in solid tumors chemotherapy is used either as adjuvant or neoadjuvant. The chemotherapy is an intense and cyclic treatment and unlike surgery has many side-effects like hair loss, nausea, vomiting, and diarrhea. Long periods of treatment, repeated hospitalizations and side-effects of chemotherapy beside the knowledge of having cancer can all affect the psyche of these patients. In context of cancer, distress is defined as extending along a continuum ranging from common normal feeling of vulnerability, sadness and fear to problems that can become disabling such as depression, anxiety and Panic, social isolation and spiritual crisis [1]. Of these, anxiety is the most commonly seen in cancer patients. It can occur in four forms i.e. situational anxiety, disease related anxiety, treatment related anxiety and as an exacerbation of pre-treatment anxiety disorder [2]. In the present study we used distress inventory for cancer version 2 (DIC 2)to measure preclinical distress [3] and hospital anxiety and depression scale (HADS) to evaluate clinical case ness for anxiety and depression in patients undergoing chemotherapy to evaluate the effect of chemotherapy in these patients and other factors that may contribute to these.

## Patients and methods

A total of 117 patients undergoing chemotherapy were evaluated for distress, anxiety and depression using DIC 2 [3] and Malayalam version of HADS. The results of Malayalam translation and validation of HADS has been published earlier [4]. After obtaining the written informed consent two of the co-authors (SGP, DN) carried out the interviews. It normally took 30–60 minutes for an interview to complete. The interviews were carried out while the patients were waiting in the day care for their chemotherapy. Personal details like age, gender, education, occupation, marital status, religion, and details of spouse and family were also collected.

Statistical analysis was carried out using one way Anova, Chi square test and Pearson's product moment correlation.

### Tools used

Distress inventory for cancer (DIC 2) is a 33 item tool which can be administered by an interview or can be self administered. The tool gives a global distress score beside subscale scores. The scale is scored on a 5 point Likert scale, however, the family specific subscale having questions on spouse and children has additional option of marking it as not applicable, if the person being interviewed is not married or does not have children. The scoring for the scale and subscale scores is being done as per the manual for scoring of DIC V2 [3].

### Hospital anxiety and depression scale (HADS)

HADS is a 14 item instrument designed to detect the presence of anxiety and depression. The Malayalam version of the tool was used. The tool has been translated using standard formers backward- forward technique and has been validated [4]. The tool is translated with permission of nferNelson, UK the copyright owner. A score of 11 or higher was considered as significant case ness while 8–11 represented mood disturbances.

### Permissions

Permission was obtained for Institutional review board and Ethics committee for the study. Written informed consent was obtained for all patients being interviewed.

## Results

The mean age of the patients were 45.4 ± 15.8 year, there were 62 (53%) males and 5 (47%) females. At the time of interview nearly three forth of the patients were married and over 50% were Hindus. Nearly 45% of the patients were poor and 31% belonged to upper social class. Of the 117, 52 (44.4%) were taking chemotherapy for solid tumor while 33 (28%) had lympho-porliferative disease and 20 (17%) had hematological malignancies. Majority of the patients 36 (30.8%) had sage III disease. Details of the patient characteristics are detailed in table 1.

**Table 1:** Patient characteristic

**Gender**	**No.**	**Percent (%)**
Male	62	53
Female	55	47
**Marital status**		
Married	57	74.4
Single/Widowed	30	25.6
**Religion**		
Hindu	62	53
Christian	33	28.2
Muslim	22	18.8
**Social status**		
Lower	52	44.4
Middle	29	24.8
Upper	36	30.8
**Stage of disease**		
I	7	6
II	32	27.4
III	36	30.8
IV	25	21.4
X	17	14.5
**Type of illness**		
Solid tumor	52	44.4
Hematological	20	17.1
Lympho-proliferative	33	28.2
Myeloma	12	10.3
**Distance traveled**		
< 150 km	67	57.3
> 150 km	50	42.7

Table 2 describes the distress, anxiety and depression scores. The mean distress scores were 24.04 ± 9.06 (range 7.14–63.6) while for distress subscales it ranged from 0.0–43.0. The mean anxiety scores were 3.33 ± 3.5 while for depression it was 4.07 ± 3.24.

**Table 2:** The distress, anxiety and depression scores

**Distress inventory V2**	**Mean**	**SD**	**Median**	**Min**	**Max**
Emotional distress	25.4	7.56	25	10	43
Spiritual distress	10.0	3.64	9	5	25
Social distress	9.23	2.79	9	6	19
Medical distress	4.39	0.78	4	4	8
Activity of daily living	2.8	1.8	2	1	5
Family specific distress	9.68	6.11	11	0	23
Total distress (DIC2)	24	9.06	22.7	7.14	63.64
**HADS**					
Anxiety	3.33	3.5	2	0	14
Depression	4.07	3.24	3	0	16

Table 3 describes the results of one way Anova. Age above 47 years was found to significantly increase spiritual distress and activity of daily living while high income and upper social class patients had less emotional, family specific and total distress. Hindus and myeloma patients were found to have significantly high spiritual distress.

**Table 3:** Mean scale and subscale scores and Results of one way Anova

**Age**	**ED**	**SPD**	**SD**	**MD**	**ADL**	**FSD**	**DIC**
<47 Years	26.5	9.5	9	4.3	2.4	12.9	24
>47 Years	24.4	11.0*	9.45	4.4	3.1*	12.2	24
**Gender**							
Male	25.5	10.3	9.5	4.4	2.7	12.8	24.7
Female	25.3	9.7	8.8	4.3	2.9	12.2	23.2
**Income**							
Low	28.3	9.7	9.2	4.3	3.2	13.8	26.9
Middle	24.1	11.0	9.2	4.6	2.6	12.6	24.1
High	22.2**	9.6	9.3	4.3	2.3	10.7*+	19.7**+
**Religion**							
Hindu	25.4	11.5	9.5	4.4	2.9	12.2	25.4
Non-Hindu	25.4	8.3**	8.8	4.3	2.7	12.8	22.4
**Marital Status**							
Married	26	10.2	9.4	4.3	2.9	__≠	24.7
Single/Widowed	23.6	9.5	8.7	4.4	2.5	__	22.0
**Type of Cancer**							
Hematological	26.6	9.1	8.4	4.4	2.7	12.5	23.8
Lympho-proliferative	25.9	8.5	9.5	4.2	2.7	11.6	22.8
Solid	24.4	10.9	9.2	4.4	2.7	13.1	24.3
Myeloma	26.1	12.0*^#^	9.5	4.5	3.2	12.2	26.3
**Stage of Disease**							
Stage I	24.4	8.7	10.2	4.0	2.1	13.5	22.8
Stage II	23.3	9.3	9.3	4.4	2.5	11.7	21.4
Stage III	24.8	11.4	9.4	4.3	3.0	11.4	24.1
Stage IV	28.4	9.8	9.8	4.4	3.1	13.5	268
**Distance traveled**							
> 150 km	25.4	10	9.2	4.3	2.9	12.0	23.6
> 150 km	25.5	10.1	9.2	4.4	2.6	13.2	24.5

+ low with high * Significance ≤ 0.05 ** Highly significance ≤ 0.001

≠ no comparison was made as score for single/widowed group was zero for this subscale.

# Lympho-prolifrative with solid tumors and myeloma.

Table 4 describes the factors influencing anxiety and depression. Proportion of patients with depression was slightly higher than anxiety.

**Table 4:** Results of factors influencing anxiety and depression.

**Age**	**Anxiety**		**χ**^**2**^	**p**	**Depression**		**χ**^**2**^	**p**
	+	-			+	-		
< 47	8	49			11	46		
			0.01	0.8			0.38	0.53
> 47	10	50			8	52		
**Gender**								
Male	10	52			14	48		
			0.05	0.81			3.8	0.04*
Female	8	47			5	50		
**Income**								
Low	12	40			11	41		
Middle	2	27	4.4	0.10	3	26	1.8	P = 0.40
High	4	32			5	31		
**Religion**								
Hindu	11	51			9	53		
			0.5	0.45			0.2	0.59
Non Hindu	7	48			10	45		
**Marital status**								
Married	13	74			16	71		
			0.05	0.82			1.1	0.28
Single/widow	5	25			3	27		
**Disease type**								
Hematological	3	17			3	17		
Lympho-proliferative	3	30			4	29		
			2.79	0.42			1.2	0.73
Solid tumor	11	41			11	41		
Myeloma	1	11			2	11		
**Stage**								
Stage I	1	6			1	6		
Stage II	4	28			4	28		
			0.6	0.89			1.6	0.65
Stage III	7	29			5	31		
Stage IV	4	21			6	19		
**Distance**								
< 150	11	56			10	57		
			0.12	0.17			0.19	0.65
> 150	7	43			9	41		

## Discussion

Chemotherapy given for treatment of cancer kills cells that are fast dividing, which is hallmark of cancer. In the process it also kills normal cells that too have a tendency to divide rapidly like cells in the bone marrow, oral cavity and mucosal lining of intestine, hair follicles, ova and sperms. Hence, the side-effects of chemotherapy are linked to these. The nausea and vomiting at times is so severe that a subsequent visit to hospital itself may produce nausea which is termed as "anticipatory nausea".

Like the present study, where a higher proportion of depression was observed in men compared to women, gender differences are also observed by other authors, though in other studies these are seen more in women. Keller and Henrich (1999) [5] found that women are more likely than man to engage in illness related behavior including perceiving and reporting symptoms, utilizing informal and health care services. It is reported that doctors take symptoms reported by man more seriously while symptoms reported by women are often interpreted as psychological ensuing higher frequency of prescriptions for psychotropic drugs [6]. Despite female patients reporting symptoms and higher overall distress due to illness compared to male patients, general satisfaction with life however did not differ significantly between genders.

Symptom occurrence, their intensity and symptom distress has been studied from time of admission to discharge in patients undergoing high dose chemotherapy and stem cell transplant [7]. The results suggested that symptom occurrence followed a curve where highest frequency of symptoms was noted from the day of transplant to the end of protective care period. These included tiredness, loss of appetite, dryness of mouth, nausea and sleep disturbances [7] most importantly patients reported to have anxiety at the beginning were found to have higher anxiety at the end. No such comparison could be made in present study due to its cross-sectional design. Trask et al (2003) [8], too showed that 30–50% of the subjects show moderate to high level of distress before the start of the treatment. Dose intense adjuvant chemotherapy regimens showed higher although transient psychological distress in early breast cancers in a study [9]. Another study found significantly higher amount of anxiety in patients receiving chemotherapy [10]. In the present study however, there was no other group to compare proportion of patients with anxiety and depression. Granisetron an antiemetic has also been found to be less effective in patients with manifest anxiety [11]. Effect of age has been evaluated and older patients and men had been found to have less anxiety and depression [12], we however failed to find any such relation in our study. Symptom anxiety and symptom experience in patients undergoing chemotherapy has been examined and significant association were found with psychological symptoms but not for visible symptoms [13], however in the present study we did not observe any relations with symptoms.

Cancer related depression is a pathological affective response to loss of normality and one's personal world as a result of cancer diagnosis, treatment or impending complications. Similar to Grief, depression presents with symptoms of sadness, fearfulness feeling of panic and yearning for lost objects [14]. Depression is suspected when symptoms of sadness persist and are accompanied by increasing dysfunction, feeling of worthlessness, lowered self-esteem, suicidal preoccupation or inability to anticipate anything with pleasure [15]. Miranda et al., (2002) [16] evaluated depression in breast and cervix cancer patients undergoing neoadjuvant chemotherapy and found no difference in proportion of depressed however, the number of depression patients increased after chemotherapy for breast cancer which is reduced for uterine cervix cancer [16]. The patients who responded to treatment were less depressed. Though the number of patients with depression in the present study was low, still the need for intervention to improve psychological morbidity cannot be ignored. Distress appears to be present in early part of continuum and may have possible overlap with anxiety. Further studies with higher sample size are needed to further elucidate this relationship.

## Conclusion

The study highlights high psychological morbidity of cancer patients and influence of gender on depression. Construct of distress as evaluated by DIC 2 may have a possible overlap with anxiety

## Conflicts of interests

The author(s) declare that they have no competing interests.

## Authors' contributions

MP: conceived and designed the study and revised the final draft of the manuscript.

GPS and ND: conducted the patient interviews, collected the data and prepared the draft manuscript.

BCT: performed the data analysis

BMH and RK: Participated in study design and revision of the mansucript
